# The Electronic Health Record Objective Structured Clinical Examination Station: Assessing Student Competency in Patient Notes and Patient Interaction

**DOI:** 10.15766/mep_2374-8265.10998

**Published:** 2020-10-28

**Authors:** E. Shen, Joseph Anthony Cristiano, Leslie Renee Ellis

**Affiliations:** 1 Assistant Professor, Department of Internal Medicine, Section of General Internal Medicine, Wake Forest Baptist Health; 2 Associate Professor, Department of Internal Medicine, Section on Hematology and Oncology, Wake Forest Baptist Health

**Keywords:** Electronic Health Record, Objective Structured Clinical Examination, Doctor-Patient Relationship, Clinical Reasoning, Standardized Patient, Communication, Clinical Evaluation, Physician, Assessment, Clinical Reasoning/Diagnostic Reasoning, Educational Technology, Informatics/Health IT, Professionalism

## Abstract

**Introduction:**

The ability to utilize the electronic health record (EHR) without compromising the doctor-patient relationship (DPR) is an essential skill of all physicians and trainees, yet little time is spent on educating or assessing learners on needed techniques. To address this gap, we developed a conventional OSCE station coupled with a simulated patient chart within the Epic program in order to assess our students' skills utilizing the EHR during a patient encounter.

**Methods:**

Of third-year medical students, 119 were given full access to the patient's simulated chart 24 hours in advance of their OSCE to review clinical data. During an in-person OSCE with a standardized patient (SP), students performed a focused history and physical, using the EHR to verify allergies and medications. Students completed an electronic patient note graded by faculty. SPs evaluated the students on communication and interpersonal skills with specific rubric elements. Faculty graded the students' notes to evaluate their expression of clinical reasoning in the assessment and plan.

**Results:**

Training SPs and faculty to assess students on EHR skills was feasible. After implementation of a comprehensive curriculum focused on EHR and DPR, there was a significant difference on EHR-related communication skills (*M* = 76.4, *SD* = 17.6) versus (*M* = 37, *SD* = 28.9) before curriculum enhancement *t* (117.9) = −12.4, *p* <.001.

**Discussion:**

The EHR OSCE station provided a standardized method of assessing students' EHR skills during a patient encounter. Challenges still exist in the technological requirements to develop and deliver cases in today's EHR platform.

## Educational Objectives

By the end of this activity, learners will be able to:

1. Apply sound doctor-patient relationship principles during an electronic health record (EHR) OSCE exam.
2. Perform a patient-centered focused history and physical exam while using the EHR in a clinical encounter.
3. Use the EHR to verify patient allergies and medications.
4. Write an electronic patient note in the EHR system.

## Introduction

The use of an electronic health record (EHR) is universal in the modern day practice of medicine. This transformation has been rapid and catalyzed by incentives included in legislation such as the 2009 Health Information Technology for Economic and Clinical Health Act and the Affordable Care Act of 2010. Accordingly, the ability to utilize the EHR is an essential skill of all physicians and trainees, regardless of the setting or specialty. Increasingly, most physicians now report using an EHR to fulfill a growing number of the core duties of their medicine practice.^1^ Understandably, this transformation in health care delivery has generated great need within undergraduate medical education for large-scale curricular enhancements and updates to address the myriad new EHR-specific skills sets essential to modern day patient care.^2,3^

The AAMC recognized the need for graduating medical students to “demonstrate patient-centered interview skills” as a core entrustable professional activity (EPA 1.0).^4^ However, use of the EHR is not traditionally included while training on the doctor-patient relationship (DPR), and the EHR poses unique challenges. Effective strategies in the use of the EHR related to the DPR is a potentially teachable skill set if there is focused attention in undergraduate and graduate medical education. Accordingly, innovations to develop such medical education curricula that incorporates the EHR into clinical care are growing. Lee and colleagues developed and published a novel educational curriculum along with the mnemonic *human level* that teaches and assesses trainee performance in EHR use, emphasizing patient-centered strategies.^5^ This curricular innovation provided simulation videos, an instructional session, and an OSCE station focused on the necessary skills to remain patient-centered while incorporating the EHR into the visit.

Beyond patient-centered communication, the complex data processing for the ever immense data available in the EHR represents an emerging skill set.^6–12^ Similarly, Biagioli and colleagues implemented an EHR OSCE exam at Oregon Health & Science University and University of Texas Health Science Center at San Antonio. Students were evaluated on both patient-centered EHR-related communication and expanded emphasis on data management skills.^7^

Yet, there is continued opportunity for medical student curricula to evolve and hone in on the growing proportion of clinical care tasks that increasingly require use of the EHR. Beyond the patient-centered use of the EHR, there is prolific attention in the literature on the impact of the EHR on the quality of clinical documentation. The Alliance on Clinical Education asserted the urgency of this need and has suggested that accrediting bodies use stronger language in their educational focus on electronic documentation to facilitate rapid development of curricula for EHR specific competencies.^6^

Accordingly, EHR use in the modern day patient encounter requires simultaneous incorporation of a multitude of new skills. There were few examples in the literature in which students were assessed on the choreography of these skills together.^7–14^

In this report, we will describe an OSCE station we developed to build on the existing work of others assessing student performance in EHR use during a patient encounter. We incorporated the assessment methods of our predecessors in patient-centered communication in the use of the EHR and performance of EHR data processing. However, in our station we also emphasized evaluating learner performance on how to the use the EHR database available for review prior to the encounter in order to appropriately focus and enhance their traditional data-gathering strategies. We also incorporated the assessment of electronic note writing assessment into our EHR OSCE station.

## Methods

### Case Development

We developed an OSCE case in which the students evaluated a patient presenting to their primary care physician for reevaluation of diabetes. In addition to the conventional patient script, we developed an electronic patient chart corresponding to the case within our institutional Epic (Epic Systems Solutions) training environment. Epic is one of the market leaders in EHR systems and it was the only EHR system available to us. However, this case can be easily adapted to other EHR systems. The environment differed from our clinical operations production version by representing similar functionality to the operational version used in our clinical enterprise, but with added ability to create simulation cases without real patient data.

In our OSCE station, the electronic patient chart built-in training environment included prior office visits, vital signs, labs, medications, allergies, and other clinical data available for student review both before and during the OSCE station. The case was intentionally created with a substantial volume of clinical data to require the student to exercise critical thinking in determining the essential information needed, reconciling information from different areas of the chart, and identify gaps in information needing clarification during the patient care visit. Expert advice from an endocrinologist was obtained in the development of all aspects of the content of the case.

### Student Training

Since this station represented our students' first ever station with EHR use during an OSCE station, and differed in format to the six other stations administered on the same testing day, asynchronous orientation and training materials were provided to students in advance to familiarize them with the EHR environment, format of the station, and grading expectations. We developed a screen capture video and made it available in the learning management system. Video scripts were provided (Appendix A) so that interested institutions can adapt them for their own implementation.

### Standardized Patient Training

Standard practice at our institution includes extensive standardized patient (SP) training (Appendix B). Scripts were provided to the SPs prior to an in-person training session held by our SP coordinator and expert (physician) faculty. SPs were expected to have memorized the script prior to arrival for the training session. This script also included information about the dataset available in the patient's electronic chart. In addition to the standard training performed for other OSCE stations, additional training was provided to the SPs pertaining to student evaluation related to EHR usage and how this might affect the DPR.

### Case Administration

The EHR OSCE station was initially implemented in the spring of 2017 to 110 third-year medical students during their comprehensive clinical practice examination (CPX) during their clerkship year. During CPX testing, students participated in six stations. The EHR OSCE was added on as a seventh station but did not contribute to the students' cumulative grade. These students did not have any prior formal training on the EHR as related to the DPR, EHR data management skills, or electronic technology proficiencies for note writing. Consequently, we considered this group as the pilot or control group. After this OSCE was administered, we made minor updates based on performance data and feedback. Subsequently, we administered the EHR OSCE the next year to 119 third-year medical students after implementing EHR-specific skill training in the clinical skills course administered in the first 2 years of medical school.

Twenty-four hours before the exam, students were given full access to the electronic patient chart with the full database of clinical information. This chart included patient allergies, medications, labs, and other diagnostic data, as well as clinical notes, and was created in the EHR using the information included in Appendix G. Students were able to review the patient chart from their laptops on or off campus, and they were allowed to write notes on a 3×5 notecard as a reminder of important information to aid in focused interviewing during the patient visit; these notecards were collected by medical education staff following the EHR station.

On the day of testing, during our standard orientation for the students reviewing the CPX testing processes, additional emphasis was provided to the students about the differences between the EHR case and the other CPX stations. For all stations, including the EHR station, students were given a case summary sheet before entering the patient room (Appendix C). Students were given 15 minutes for the patient encounter.

After the patient encounter, students were given 10 minutes to complete an electronic note in the same format (Appendix D) as utilized on the other non-OSCE stations. During the pilot phase, the students completed their documentation within the ACE7 environment, but in the full-scale implementation documentation was performed in LearningSpace to maintain consistency with evaluation software used for documentation in the other stations.

### Case Evaluation

The SP checklist included standard questions about what history and physical exam questions they were asked by the students, as well as an assessment of their communication and professionalism (Appendix E). Additional questions were added to evaluate EHR-specific DPR skills. These questions focused on determining student performance in their ability to maintain a humanistic approach with balanced use of the EHR and maintaining focus on the SP rather than the EHR, particularly during any emotionally sensitive moments in the interaction.

During the 2017 administration, all SP questions on all OSCE stations related to communication and professionalism were based upon the Likert scale (*excellent*, *very good*, *good*, *fair*, and *poor*). However, to provide more clear guidance for the SPs in their assessment, in 2018 we provided descriptive anchors for the entire evaluation instrument, including those related to EHR-associated DPR skill assessments. For the SP history scores, we calculated the average SP evaluations both before and after our instrument was revised.

Clinical documentation was evaluated by the OSCE station clinical faculty who developed the case. Students were advised that their electronic documentation evaluation emphasized assessment of key elements of history, physical exam and diagnostic workup, and therapeutic plan. The evaluation tool also included elements of documentation of their clinical reasoning, which was categorically different compared to our other OSCE stations (Appendix F).

## Results

Overall, the students performed well on EHR-related communications skills based on the SP evaluation (Table 1). Only 10% of our SPs felt ignored or that the computer took priority at the beginning of the visit. SPs reported that a total of 70% students “primarily focused on my concerns and emotions but on occasion seemed more focused on the computer,”(26%) or were “able to address my emotions and concerns without being distracted by the computer”(44%). SPs also reported that a total of 79% were were *very familiar* (29%) or *mostly familiar* (50%) with the patient history. Lastly, SPs reported that 58% of students “spent a little bit of time on the computer and did so in a way that enhanced our communication and understanding my story.”

**Table 1.**
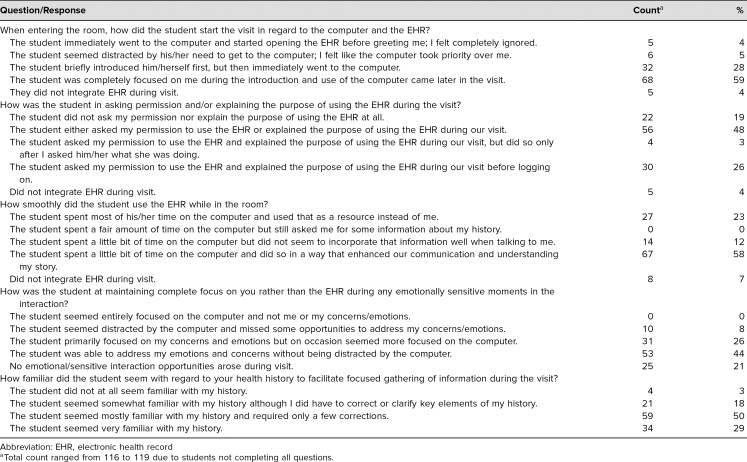
SP Evaluation of Student Performance on EHR-Related Communication Skills (*N* = 119)

Compared to the pilot administration in 2017, where students had no instructional preparation, the students who participated in the EHR-specific curricular enhancements in 2018 performed better. Performance on all five questions related to EHR communication skills was higher in 2018 with an average score of 76.4 (*SD* = 17.6) compared to 37 (*SD* = 28.6) in 2017. An independent sample *t* test revealed that the difference is statistically significant: *t* (117.9) = −12.4, *p* <.001. This illustrated that teaching the EHR curricular enhancements was impactful and effective in OSCE performance.

The average scores and standard deviation for the EHR OSCE station compared to the six non-EHR OSCE stations are shown in Table 2. The SPs rated student overall communication for the EHR station (*M* = 69.1) similarly to the non-EHR OSCE stations (*M* = 72.2), but the patient note scores for EHR station OSCE (*M*_history_ = 48.1, *M_physical__exam_* = 53.6, *M_workup_* = 30.1)were significantly lower (*p* <.01) than the average scores of the other traditional OSCE stations on all comparable categories (*M*_history_ = 75.0, *M_physical__exam_* = 66.2, *M_workup_* = 63.4).

**Table 2.**
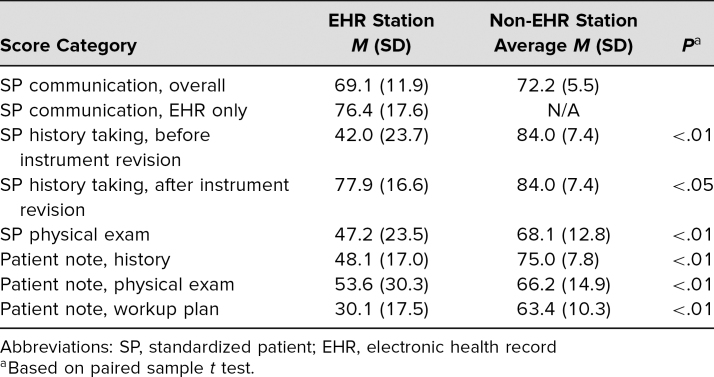
Comparison of SP Evaluation of Students on EHR OSCE Station and Non-EHR OSCE Stations

## Discussion

We ultimately had two goals with the development of this case: first and foremost, to develop and implement an assessment intervention to serve the unmet need of evaluating our students' capabilities in incorporating the EHR into a patient care encounter, and secondly, to determine whether or not the curricular enhancements added in the clinical skills course was impactful in EHR proficiencies. The OSCE was developed in Epic, but can be easily adapted to any other EHR systems.

### SP Communication Skills

Performance of the traditional non-EHR DPR skills was similar to the other OSCE stations. This was expected since those skills are emphasized so heavily throughout the entirety of the medical student curriculum and thoroughly introduced and reinforced through the 2-year clinical skills course in the preclinical years.

However, until curricular updates were implemented in 2018, students were not exposed to the use of the EHR in clinical skills and consequently would have to acquire those skills without instruction, practice, or deliberate feedback once immersed in the clinical setting. Accordingly, we hypothesized during our pilot phase that students would not have developed proficiency in the use of the EHR in a way that builds on rather than diminishes communication skills and more broadly the DPR.

Our findings supported that the implementation of EHR-specific enhancements to address its role in the DPR were effective in the acquisition of this skill set. We developed preclass asynchronous instructional materials and videos to support the flipped classroom model related to this skill set. We incorporated role-playing and both peer and faculty feedback during class for the EHR skill set.

Based on comparing our 2017 preimplementation to 2018, there was a statistically significant improvement in performance in the EHR-specific communication skills as evaluated by our SPs. This was consistent with the impact of other educational innovations that showed that this skill set is teachable with the use of effective educational strategies that incorporate learner engagement and practice of such skills. We believe our methods were complementary and added to the body of knowledge that currently exists. Specifically, our assessment instrument allowed for evaluation of how reviewing a patient's chart prior to the OSCE station could potentially enhance the students' ability to obtain a focused history. This is very similar to real-world medicine in which physicians would be able to preview a patient's electronic chart prior to the visit. Although not directly evaluated, we believe that students who were familiar with the patient's existing medical record not only were more effective in gathering a focused history, but also in building rapport with the patient. Additionally, although only minimally evaluated in our OSCE, we believe that considering the modern-day time constraints of clinic practice, familiarity with information available within the EHR will allow for building a richer history or spending more time on shared decision making.

### History and Physical Exam

The patient history gathering scores in the EHR station were lower than in the other OSCE stations. We felt the lower score in this station compared to the other OSCE stations related to the necessity of a shift in history-gathering skills required for this station compared to the others. In particular, the history-gathering expectations in this station focused around taking a brief history of the status of a chronic health problem as compared to an acute symptom evaluation traditionally emphasized throughout medical education. Additionally, this OSCE station required critical thinking in disease-to-disease interaction. Again, the critical thinking skills needed in such an evaluation differed from that of the evaluation of the other acute symptoms' stations. We did anticipate that this shift in the framework may be confusing to students, and in our orientation information we emphasized this difference. However, given that medical school curricula does not iteratively provide curricular feedback to students on this area of clinical reasoning, we suspected that test anxiety from lack of familiarity may have been a factor.

Students also scored significantly lower on the physical exam for the EHR station than on the physical exam for the other OSCE stations, as assessed both by the SP during the encounter, as well as by the faculty based upon the documentation in the note. We felt this was multifactorial. We suspected that students were unfamiliar with performing an exam in the setting of the evaluation of the status of a chronic health condition rather than an acute problem. Additionally, we believe the time-constraints associated with the added EHR tasks may have contributed.

### Assessment of Note Writing and Clinical Reasoning

Students performed significantly lower in note writing related to the assessment and plan. We believe the greater expectations related to the expression of clinical reasoning in the assessment and plan was a central factor as compared to the other stations that followed a very similar format to the Step 2 clinical skills examination format. Additionally, we felt students may not be as familiar and comfortable at this stage of their training with the assessment of a chronic health condition and the integration of disease-to-disease interactions in their clinical reasoning. Lastly, we felt that although students were taught the pharmacological approach to diabetes management, this may not be a skill regularly practiced up to this point in their training.

Interestingly, we noticed that students seemed to focus on recommending dietary changes for this patient, which was not within the scope of their knowledge that we wanted to assess; future iterations of this script included our SPs informing the students that they were meeting with a personal trainer and nutritionist after their meeting with the student, to help redirect the students away from spending the majority of their time with the patient counseling them about diet and exercise. Again, improved communication about our expectations for this station will likely improve student scores. However, this station also may identify an area of need in our broader medical education curriculum to emphasize clinical reasoning and decision making in chronic disease management.

### Instrument Development

In reviewing the feedback from SPs during our training and after the OSCE, the anchored communication and professionalism questions allowed our SPs to provide more accurate assessments of our students in relation to general communication and professionalism skills as well as those related to EHR usage during the patient encounter. Continued training was a must to make sure the SPs were utilizing the forms appropriately and for clarifications to be made.

### Feasibility

Overall, we successfully implemented an EHR OSCE case for an entire class of students. The development of the SP communication, physical exam, and history checklists made it possible to implement the EHR OSCE station on a large scale and without a great amount of time required by faculty for grading. During the case development stage, close collaboration between physicians and the Epic ambulatory training consultant was one of keys to the success of this project. Securing senior leadership support both on case development and implementation was another important step we took to ensure gains in this endeavor.

Our data demonstrated the importance of EHR communication skill training in preclinical years. Students who received the EHR communication training drastically outperformed those students who did not receive the training.

This OSCE station could be easily implemented at peer institutions in the undergraduate or graduate medical education setting. Ideally this case should be implemented for students who have had experiences with the EHR during their clinical years. At our institution, the case was introduced along with other cases during a CPX exam that aimed to prepare student for the Step 2 clinical skills exam. Because the format of the EHR OSCE case was different than other EHR cases, careful planning was required for the success of the OSCE station. The significant discrepancy in scores between students' performance on the EHR station as compared to the other OSCE stations were likely related to suboptimal communication on our part of what other goals we were trying to achieve with this EHR station.

### Limitations

There were limitations associated with our EHR OSCE station. First, our EHR OSCE was implemented during a testing day with other OSCE stations that differed in format and assessment methods. Due to the focus on the EHR in our station, the OSCE SP and documentation grading rubrics were modified to incorporate the EHR skills being assessed. Despite diligent communication in advance, we believe that the differences in the OSCE station may have influenced learner performance. Additionally, we acknowledge that our students were not routinely and systematically evaluated in our curriculum for chronic disease management related to diabetes. However, our students performed this OSCE station after the completion of their third-year clerkships and we felt by focusing on diabetes management we could more realistically simulate the longitudinal chart review data gathering skills emphasized in our assessment methods. Lastly, we acknowledge that students were required to complete additional EHR-specific tasks during the encounter and it imposed a time constraint. However, we believe that giving students 24 hours to preview the patient's electronic chart allowed them ample opportunity for data processing and consequently gave them the opportunity to enhance their in-room data gathering efficiency.

### Future Directions

This OSCE case can be utilized as a stand-alone, high-fidelity simulation case in an ambulatory clerkship such as ambulatory internal medicine to assess student EHR-related communication and patient note skills. As EHR adoption rate has been increasing and early evidence of such adoption is having a positive impact on patient care, it is imperative for us as medical educators to create curricular and assessment tools to teach and measure future physicians' EHR-related communication and patient note skills. In this submission, we presented an early attempt to do so. We call on our peers to help develop a library of EHR cases that can be shared among institutions.

## Appendices

EHR OSCE Introduction Video Script.docxOSCE SP Training Guide.docxOSCE Exam Case Summary Sheet.docxOSCE Patient Note Template.docxOSCE SP Postencounter Checklist.docxOSCE Patient Note Faculty Grading Rubric.docxEHR SP Case.docx
All appendices are peer reviewed as integral parts of the Original Publication.
