# Supplementary material for: The Electronic Health Record Objective Structured Clinical Examination Station: Assessing Student Competency in Patient Notes and Patient Interaction
Source: MedEdPORTAL. 2020 Oct 28;16:10998. doi: 10.15766/mep_2374-8265.10998 (PMC7597945; doi:10.15766/mep_2374-8265.10998)
Supplement: Supplementary file 1 — EHR OSCE Introduction Video Script.docxOSCE SP Training Guide.docxOSCE Exam Case Summary Sheet.docxOSCE Patient Note Template.docxOSCE SP Postencounter Checklist.docxOSCE Patient Note Faculty Grading Rubric.docxEHR SP Case.docx [file mep_2374-8265.10998-s001.zip › A. EHR OSCE Introduction Video Script.docx]

**Appendix A - EMR OSCE Instruction Video Script**

Hello, my name is _________ and I am the _________. This training is for third year medical students who will be completing the CPX EMR project. It will outline instructions on how to access the appropriate WakeOne training environment and complete the project. Let’s get started.

From your desktop, locate and double-click the ACE7 WakeOne icon. Using the project tent card you received, log in with your User ID and password. Select OK to accept the default department that populates, PPI 03 FAMILY MED MODULE B.

Note: For the purposes of this project, you are logged in as an attending provider.

The day before your patient’s appointment, you will be expected to review labs and documentation from prior visits in WakeOne. Select the Schedule workspace to get a full view of the outpatient schedule. Click once on the schedule folder with your name to view appointments scheduled with you only. Because your Sally patient has not yet arrived for her appointment, we should not double-click on her row as this will open the encounter. Instead, click once on her name and select Review from the Schedule toolbar to launch Chart Review. From the Encounters tab, review her past visit from 9 months ago, 6 months ago, and 4 days ago. These reports will provide the visits’ vitals, progress notes, and etcetera.

To review Sally’s labs, select the Labs tab of Chart Review. Take note of the POCT Glucose and Hemoglobin A1c results. You can also review labs from the Results Review activity tab.

The day of your patient’s appointment, access your outpatient schedule again and double-click to open Sally’s chart. The Visit Navigator opens by default. This navigator’s purpose is to streamline your visit documentation from top to bottom. For the purposes of this project, we will strictly look at the sections required for closing an encounter.

Open the Allergies navigator section. Add any agents not currently documented if needed, and then click Mark as Reviewed.

Navigate to the Medications section. After verifying this list is up to date, select a Med List Status of Provider Reviewed and click Mark as Reviewed.

Next, open the Progress Notes navigator section and click Create Note. A SmartText template has been created for this project. In the Insert SmartText field, type “cpx” and ENTER. Select the CPX PROGRESS NOTE SmartText template. After completing your note, leave “Sign at Close Encounter selected and click Close to see this section in a view only format.

Move on to the Visit Diagnosis navigator section. The Visit Diagnosis is the primary reason for the patient’s clinic visit. Upon entering a diagnosis, you may be required to enter greater specificity within the Diagnosis Calculator to create an ICD-10 code.

Lastly, open the Sign Visit section. You receive a hard stop indicating that a level of service has not been entered for this encounter. Click the hyperlink within this hard stop warning; the LOS navigator section opens. Select a NO EM level of service.

Return to the Sign Visit section. For the purposes of this project, it is okay if you still have recommended items. Click Sign Visit. Sally’s encounter is now closed.

This concludes this training. After closing your patient’s encounter, you will move on to the next station of the CPX EMR project.
