# Supplementary material for: The Electronic Health Record Objective Structured Clinical Examination Station: Assessing Student Competency in Patient Notes and Patient Interaction
Source: MedEdPORTAL. 2020 Oct 28;16:10998. doi: 10.15766/mep_2374-8265.10998 (PMC7597945; doi:10.15766/mep_2374-8265.10998)
Supplement: Supplementary file 1 — EHR OSCE Introduction Video Script.docxOSCE SP Training Guide.docxOSCE Exam Case Summary Sheet.docxOSCE Patient Note Template.docxOSCE SP Postencounter Checklist.docxOSCE Patient Note Faculty Grading Rubric.docxEHR SP Case.docx [file mep_2374-8265.10998-s001.zip › B. OSCE SP Training Guide.docx]

**Appendix B - OSCE standardized patient training guide**

**Sally Martin Overview**

You are a 43 year old woman who presents to your Family Medicine Clinic for a diabetes management follow up appointment.

**Your opening line:**

“I’m here for a follow-up on my diabetes management”

**How you will present yourself to the student:**

- You are friendly, however you will express concern about your health
- You are feeling overwhelmed about your recent health set-back and with all the additional medications that were prescribed (for your “cold” – information below)
- You are also overwhelmed about the increase in blood sugar readings, recently (information below)
- You are compliant with all your medications, but you really do not have a strong understanding of your diabetes management

**Diabetes history:**

- Your PCP diagnosed you with Type II diabetes approximately 3 years ago. Took an oral medication for two years, but it wasn’t working. If asked, you don’t remember the name, but it started with an “M”. If the student says “Metformin” – you say “yes”.
- Your PCP started you on insulin 9 months ago at 30 units twice daily.
- You attended a follow-up appointment 6 months ago when your PCP increased your insulin 40 units twice daily.
- You missed your follow up appointment 3 months ago due to a “fender-bender” on your way to the clinic (nobody was hurt, but the front wheel was too damaged to drive on).
- You are currently taking 40 units of insulin twice a day.
- Your blood sugar readings have normally been 160 to 200, but recently they have been in the 240-260s. You will wonder if being sick and being on the medications you were prescribed for your “cold” are the reason. **** Do not volunteer to student****

| **Diabetes Timing** | **Treatment** | Dose |
| --- | --- | --- |
| Diagnosed 3 year ago | Oral medication – started with an “M” | Don’t know |
| 9 months ago | Started Insulin | 30 units 2x day |
| 6 months ago | Insulin increased | 40 units 2x day |
| 3 months ago | Missed appointment |  |
| Today | Insulin – same dose for past 6 months | 40 units 2x day |

| **Blood sugar** | **Treatment** |
| --- | --- |
| Normally | 160-200 |
| Past several days | 240-260 |
| Goal | Less than 140 in the morning |

**Recent illness medical history:**

You were seen at the Family Medicine Clinic **4 days ago** because of a cough, fever (99 or 100 degrees) with chills and increased trouble breathing. You were sick for approximately a week before you came to the clinic. The urgent care doctor (not your PCP) prescribed an inhaler and two other medications for your “cold” (upper respiratory infection). These medications are about to be finished – but you “can’t remember if it’s tomorrow or the day after”.

**Your concerns for today’s appointment:**

- You are concerned about your diabetes and you want to discuss the diabetes management plan. **This is your priority**
- If asked about your diet, you will be meeting a diabetes educator, later today, to work on diet changes – you want to better manage your diet and diabetes – you are excited about this.
- If asked about exercise – you have signed up for a hospital-sponsored “couch to 5K walk” program. You start “training” this coming Saturday. You are excited about this, too.
- Regarding your “cold” - you are feeling much better since you started the medications 4 days ago. If the student persists in reviewing the “cold” symptoms and medications, politely re-direct him/her back to the diabetes management, as you are feeling much better.
- Regarding your diet and exercise – you have your plans – again, politely re-direct a student who persists in talking about this
- Yes, you smoke, but you want to discuss your diabetes management plan – not talk about smoking cessation plans – politely re-direct back.

If you have to politely redirect the student repeatedly, please reflect this on the Pro/Com checklist (item #2 – respectful treatment).

**Your current roster of diseases**

- Diabetes
- “COPD” from smoking
- High blood pressure
- “Kidney Problems” from diabetes and high blood pressure

**Your understanding of the roster of medications you are taking currently**

- You have been taking insulin to manage your diabetes. You are taking 40 units twice a day – that was the dose that was prescribed 6 months ago. If pressed, you’ll mention that you’re not quite sure if it matters if you take the medication before or after you eat but most of the time you are taking the insulin after eating.
- You recall that you are on 2 new medications that are temporary for your “cold.” You realize that those medications are about to be finished but not quite sure if it is 1 or 2 more days (“tomorrow or the next day”).
- You cannot immediately recall the names of your “regular” medications for COPD and kidney disease. However, if the student can remind you of the names, you are familiar with them – just not off the top of your head. You do not know the dose you are taking for any of the medications.
  - - Albuterol as required (prescribed as a “rescue inhaler” for COPD and also used for the cold)
    - Fluticasone-salmeterol inhaled twice day 1 puff (for the COPD)
    - Tiotropium inhaled once a day one puff (pill crushed in inhaler) (COPD)
    - NPH/regular insulin 70/30, 40 units twice a day with breakfast and dinner (Diabetes)
    - Losartan (kidney problems and high blood pressure)
    - Simvastatin (cholesterol)
    - Azithromycin antibiotic added at urgent care visit for your “cold” (finishing tomorrow or day after)
    - Prednisone (oral steroid) added at urgent care visit for your “cold” (finishing tomorrow or day after)

**Recent associated symptoms:**

- You have been getting up in the middle of the night to urinate (1-2 times a night)
- You have been urinating every 5-6 hours during the day. **So, yes, you are “peeing more than usual”**
- You have been more thirsty over the past few days, as well
- You have not felt “yourself” in weeks.
- Your appetite has been a bit “off” and it’s getting better now but you’ve skipped lunch a couple times and been eating more throughout the day snack foods like crackers and chips. You’ve been drinking mostly water but occasionally Coke (Regular Coke) and unsweetened tea.

The students will desperately want you to quantify exactly how much you are drinking. You don’t know exactly – but you just know you’re drinking more fluids.

**If the student uses the following words:**

- Polydipsia or Polyuria. You don’t understand those words “I don’t know what that means”. They should rephrase to: are you drinking a lot of water (or fluids) or are you peeing or urinating a lot.
- Hypoglycemic symptoms or hypoglycemia. You don’t understand what is meant by “hypoglycemic” or “hypoglycemia”. The student should rephrase to: low blood sugar symptoms or a low blood sugar event. You have never had either – your blood sugar levels have always been high not low.
- Orthopnea. This is a fancy word for having breathing issues when lying flat. You have never had this.
- The student will receive credit on the checklist for asking (if it’s on the checklist) – but, should be docked on the professionalism/communication checklist (item #7) for appropriate vocabulary.

**CASE-SPECIFIC INFORMATION**

1. **Patient Profile**
   - 43 y/o woman
   - Affect (Mannerisms, Behavior): friendly, calm but concerned about her diabetes management and increase in blood sugars. Is somewhat overwhelmed about recent illness and increase in number of medications recently prescribed
   - Social History/Lifestyle: Married and lives with husband and daughter. Son is married and has a daughter (this is the grandchild who gave her the cold that resulted in her visit to the clinic 4 days ago)/Sedentary - does not exercise – but has signed up for a hospital-sponsored “Couch to 5K walk” program
   - Occupation: Office clerk
   - Sexual History: Monogamous
   - Habits: Smoker 1 pack per day for 27 years
   - General Appearance: well appearing, middle age slightly overweight (we will provide you with a picture of the abdomen to present to the student during the physical examination)
2. **History of Present Illness for Elevated Blood Sugar:**
   - Onset: in the past several days blood sugar is 240 to 260, prior to the illness - blood sugars were around 160 to 200.
   - Duration: Blood sugars have still been elevated since her recent office visit 6 months ago, but even prior to the illness they were not at her blood sugar goal (less than 140 in the mornings).
   - Frequency: you take your blood sugar typically in the early morning before breakfast, but periodically you’ll take it mid-day (this is lunch time). Blood sugars in the mornings are usually around 240 and the mid-day readings are typically slightly higher around 260.
   - Setting: Since the onset of a recent respiratory illness about a week ago, you have noticed the increase in blood sugar
   - Aggravating/Alleviating Factors: You have been taking prednisone (a steroid) since an office visit **4 days ago** for a respiratory infection and you’ve noticed your blood sugars increase since then. Prior to that time, your blood sugars in the morning were typically 160-180 and around 200 mid-day.
   - Associated Symptoms: Waking up in the middle of the night less so due to coughing now, but rather to urinate. You have been thirsty over the past few days as well and have not felt “yourself” in weeks.
3. **History of Present Illness for Recent Respiratory Symptoms (only if asked and not crucial to data collection for today’s visit)**
   - Quality: Approximately 10-14 days ago the patient noticed increased cough and difficulty breathing; difficulty breathing particularly with any walking or exertion. These symptoms have improved progressively since her urgent care office visit 4 days ago
   - Quantity/Severity: Symptoms peaked within 1 day of her office visit but are now progressively improving. She feels her respiratory symptoms are nearly resolved now.
   - Duration: Onset approximately 10-12 days ago
   - Frequency: Symptoms are overall improving. She still has a cough but it is only seems to happen a couple of times a day. It seems to improve once she falls asleep and she does not wake up with coughing attacks any more.
   - Setting: Patient thinks her blood sugars are high due to the steroids she is taking for her “cold”.
   - Aggravating/Alleviating Factors: Cough was made worse by lying down and shortness of breath worse with exertion. Prior to recent urgent care appointment she was using albuterol approximately 6/day and it helped temporarily with cough and shortness of breath. Since the office visit patient has noticed significant improvement with oral prednisone (steroid) and she is now only using her albuterol about 1-2/day.
   - Associated Symptoms: At onset no fevers, but around the time of the urgent care appointment the patient developed fevers to 99-100 degrees. She has noticed no further fevers since her office visit. There was also associated fatigue and poor sleep (frequent nighttime awakenings with cough) initially. She is still suffering from fatigue because of continued nighttime awakenings to urinate rather than due to her cough.
4. **Past Medical History**
   - General State of Health: The patient is sedentary and recognizes that there is area to improve her health (hence the ‘couch to 5K’ program and getting a diabetes educator’s appointment to restructure her diet). Specifically, she is concerned about her diabetes and she suspects it is not well controlled. Adding to this, she is concerned about her recent respiratory infection. Prior Illnesses or Injury:
     1. Please see above for further characterization of the recent respiratory illness treated with an inhaler and 2 medications.
     2. Beyond this, the patient recognizes that she has diabetes.
     3. She also has been told she has “kidney problems” from her diabetes and high blood pressure as well as “COPD” from smoking.
     4. She does not really understand what her doctors mean by these diagnoses of “kidney problems” and “COPD” but she recognizes that there is no treatment directly for her kidneys and that her COPD is being treated by inhalers for chronic management as well as medications for her recent respiratory infection. So, in other words, she knows she has kidney and COPD issues – but **not the specific details**.
     5. Regarding her diabetes, she has some trouble with keeping her blood sugars under control. She acknowledges that her blood sugars have been significantly higher since the onset of her respiratory infection BUT they were also high previously too. She missed an appointment to re-assess her diabetes about 3 months ago and is worried because her doctor emphasized that her blood sugars were not at goal prior to that visit. The dose of insulin that she is taking is the dose that was prescribed at her first follow up visit with her PCP 6 months ago; she was started on insulin three months prior to that visit.
   - Past Hospitalizations: For birth of son and daughter.
   - Past Surgery: Laparoscopic cholecystectomy (gallbladder surgery) in 2012.
   - Allergies and Immunizations: sulfa medications cause rash with hives
   - Current Medications:
     1. Albuterol as required (prescribed as a “rescue inhaler” for COPD and also used for the cold)
     2. Fluticasone-salmeterol inhaled twice day 1 puff (for the COPD)
     3. Tiotropium inhaled once a day one puff (pill crushed in inhaler) (COPD)
     4. NPH/regular insulin 70/30, 40 units twice a day with breakfast and dinner (Diabetes)
     5. Losartan (kidney problems and high blood pressure)
     6. Simvastatin (cholesterol)
     7. Azithromycin antibiotic added at urgent care visit for your “cold” (finishing tomorrow or day after)
     8. Prednisone (oral steroid) added at urgent care visit for your “cold” (finishing tomorrow or day after). This medication has worsened your blood sugars.
   - Other Drugs: None.
   - Eyes: No issues that you have noticed. If asked – you will deny a history of blurry vision.
   - Neurologic: Before starting insulin, you noticed some numbness in the bottom of your feet and burning. It seems to be stable since you started insulin.
   - Breasts: No exam is required as part of this visit.
   - Respiratory: you have a chronic morning cough that usually clears after a few minutes in the morning. You have been coughing more frequently with a recent respiratory infection but as characterized above it is improving and you are nearly back to your “baseline”
   - Cardiovascular (including peripheral): You deny any shortness of breath lying down. You do not notice leg swelling or weight gain recently.
   - Gastrointestinal:
     1. Your appetite has declined with the respiratory illness.
     2. You deny any abdominal pain, diarrhea, blood in stool or tarry/dark stools.
   - Genitourinary:
     1. You have experienced increasing urination since you started steroids. You wake up at night 1-2 times to urinate and you urinate every 4-6 hours during the day.
     2. You deny any symptoms of retention of urine after voiding.
     3. There is no “burning” when urinating
   - Musculoskeletal: You feel a little weaker overall since getting sick.
   - Psychiatric: You are nervous about your health but your mood is positive. You deny any depression, Suicidal Ideation or Homicidal Ideation
   - Hematologic: You deny any easy bruising, bleeding or any other symptoms.

About your A1C:

- - 6 months ago was your last A1C and it was 11.0, which is high
  - You do recognize that having a high A1C means your blood sugars are not well managed.
  - Since you missed your last appointment (3 months ago), you did not get your A1C tested
  - Also recognizing that your blood sugars have not been as low as you would like, you might still have a high A1C, which is another reason you are going to see the diabetes educator to get on a diet plan.

1. **Family History**

| **Relationship** | **Age** | **Health** | **Cause of Death** |
| --- | --- | --- | --- |
| Mother | 69 |  | Alive healthy with Diabetes |
| Father | 74 |  | Alive healthy with Hypertension |
| MGM | deceased |  | Diabetes poorly controlled and started dialysis at 59. Died 68. |
| MGF | Deceased |  | Traumatic death from MVC in 60s. |
| Sister | 45 |  | Alive healthy but suffers from Diabetes |
| Brother | 41 |  | Alive healthy but overweight and has “borderline” Diabetes |
|  |  |  |  |

1. **Scenario Development**
   1. **Why are you seeing the physician?**
      1. **Chief Complaint:** “I’m here for a follow-up on my diabetes management”
   2. **How will you look and act?**
      1. You are concerned about your health. You worry that your doctor will again say something to suggest you need to improve your health. You have been “trying” to make lifestyle adjustments and take your prescribed medications but your recent respiratory infection has been a setback. Having said that, if asked, you have not stopped smoking as requested by your doctor, you have not started an exercise program – not even walking, and you have not lost any weight.
      2. You have an established relationship with your doctor. You trust him or her and it seems they truly want to help you. So you come into the appointment with established rapport.
      3. You are friendly and comfortable sharing information with your doctor.
   3. **What are your *concerns* regarding your understanding of the problem?** Having to take steroids has been discouraging because you know it is worsening your diabetes and your blood sugars are higher. You also are concerned partly since your new PCP who you established care with 9 months ago emphasize the need to better control your diabetes. You also recognize that you need to quit smoking but there has been so many other things going on between your health specific to diabetes, your recent illness and home/work that you have not been able to address this.
   4. **How will you respond to different interviewing styles?** You appreciate a humanistic style in which the doctor communicates clearly with you and demonstrates empathy and understanding. An empathic and attentive doctor will help you to build rapport during the visit. In this case, you will voluntarily provide a narrative script of the illness. IF the doctor seems rushed or too focused on data gathering or is asking primarily closed ended questions, you will too “shut down” some and simply answer their questions.
   5. **Which of your questions should “trigger” a response from the doctor?**” Your questions or suggestions as to the concern you have about your health should trigger an empathic moment---this can be in the form of legitimizing, acknowledging, understanding, etc. You are looking for the doctor to recognize the challenges you face in your health. You are hoping for some reassurance or continued partnership.
   6. **What *challenges* will you present to the doctor?** There is much that has unfolded in your health in the past several months. You are seeing your primary care physician so you “expect” that they will be apprised of your health history in general. It is certainly understandable if the doctor asks clarifying questions to remind him of your longitudinal symptoms related to your chronic health conditions but you will be a bit discouraged if they seem to unfamiliar with your health in general. You are a bit more understanding, if the student has a multitude of questions from your urgent care visit, because you saw a different provider. Also, you notice that during this visit that your physician is potentially using the computer while interviewing you. This is okay as long as the physician remains primarily focused on maintaining eye contact, attentiveness and is asking open ended questions. Some degree of divided attention between you and the computer is understandable but if the physician becomes more focused on the computer and is not paying attention to you, you will be disappointed and you will not as freely share information.
   7. **Will you ask any questions that might *distract* from the intended challenge of the case?** There is much that has happened in your health recently. Please remain organized in your responses and do not become tangential with the recent respiratory illness unless the doctor makes a deep dive into this. You will remain focused on your diabetes as detailed in the HPI and the impact of your recent illness on your diabetes. You are also mindful of the “triad” between you, the doctor and the computer. You certainly are understanding of the use of the computer if it is a helpful aid to obtain or verify information. You will be pleased if they utilize the EHR in such a way as sharing the screen with you to show some of your health information to further your understanding and insight.
2. **Physical Exam Results** (see VIII, **Physical Diagnosis Exam Guidelines**)
   1. **General Appearance:** Vital Signs to be provided to student prior to him or her entering the room.
      1. BP: 152/85 mmHg
      2. P: 60 BPM
      3. Temp: 98.7F
      4. Resp: 16
      5. POCT blood sugar: 288
      6. SpO2: 98% RA
   2. Gastrointestinal: normal
   3. Skin: No skin problems.
   4. Genitourinary: normal. You have been urinating more frequently – but there is no pain on palpation of the bladder and, if asked, you went to the restroom after you checked in. Urgency – you have had no accidents – you can get to the bathroom in time.
   5. Eyes: normal
   6. Hematologic: normal
   7. Breasts: No exam is required as part of this visit
   8. Musculoskeletal: normal muscle tone and bulk
   9. Respiratory: clear to auscultation, end expiratory wheezes at the apices bilaterally (card to be presented to student if he or she listens to the lungs above the shoulder blades)
   10. Peripheral Vascular: normal
   11. Cardiovascular: normal
   12. Neurologic: stocking paresthesia in lower extremities bilaterally in the feet (SP will portray)
   13. Mental Status Exam: alert, oriented, conversant, no deficits
3. **Physical Diagnosis Exam Guidelines**
   1. **Key Points on Physical Examination:**
      1. End expiratory wheezing in apices (card to be presented if the student listens to upper lungs)
      2. Stocking paresthesia to the feet bilaterally (SP will portray – we will train you)

**Diagram for heart auscultation: The student MUST listen to all four sites to receive point**


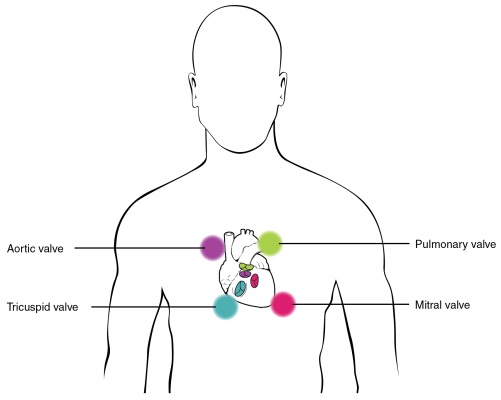


**Stethoscope Placement for Auscultation**, Image in Anatomy & Physiology, Openstax textbook website URL <https://openstax.org/books/anatomy-and-physiology/pages/19-3-cardiac-cycle#fig-ch20_03_04>, Access for free at <https://openstax.org/books/anatomy-and-physiology/pages/1-introduction> retrieved on 9/23/2019. This file is licensed under the [Creative Commons Attribution License 4.0 license](https://creativecommons.org/licenses/by/4.0/)

**
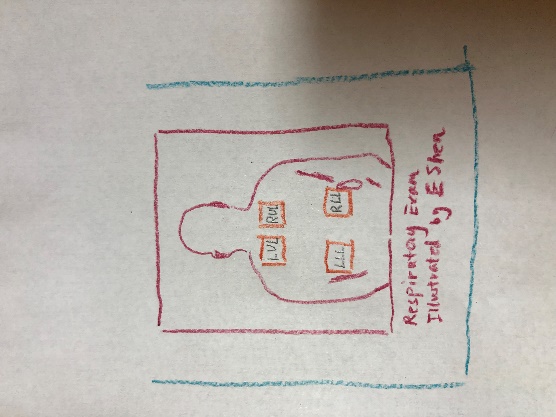
Respiratory exam: The student MUST listen to all four sites AND listen for inspiration and expiration (breathe in and out) to get credit for performing the complete respiratory exam. IF the student listens ONLY to the two upper sites – he or she does not receive a point for performing the physical exam maneuver correctly – but the student will receive the respiratory result card.**

Author (E Shen) owned

**Stocking paresthesia of the feet bilaterally (Please demonstrate testing during training).**

| 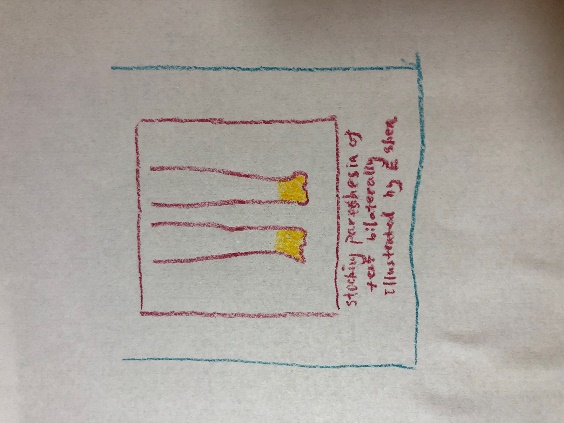  Author (E Shen) owned | **-Yes, feels the same (thighs to below calf muscle)**  **- No, this does not feel the same – it feels decreased (from just above the ankles to the toes)** |
| --- | --- |

**SP History and Physical Examination Checklist**

**Attention: No partial credit will be given. Unless otherwise specified in the bracket, check “No” if partially done.**

**History:**

Did the student elicit subjective information and/or confirm Electronic Health Record data regarding:

| 1. Recent blood sugar readings |
| --- |
| 1. Polyuria, polydipsia, or increased thirst [student receives credit if asks 1 of 3] |
| 1. Change in appetite or diet [student receives credit if asks 1 of 2] |
| 1. Hypoglycemic symptoms |
| 1. Confirm insulin dose and timing |
| 1. Confirm oral steroid (prednisone) date of end of prescription |
| 1. Upper respiratory symptoms associated with recent Urgent Care Visit |
| 1. Past Medical History/Health |
| 1. Confirm patient’s other medications against Electronic Health Record |

**Physical Exam:**

Did the student perform the following maneuvers?

| 1. Washed hands before touching patient |
| --- |
| 1. Auscultation of heart sounds |
| 1. Auscultation of breath sounds (student receives respiratory card) |
| 1. Peripheral sensory neurologic exam |
| Student used stethoscope directly on the skin for **both** respiratory system and heart sound auscultations |

**Y3 CPX PROFESSIONALISM/COMMUNICATION CHECKLIST**

Please choose the option that best describes how you feel toward the medical student’s communication skills. Some items also have a ‘not applicable’ option. Select this option when the context of the case does not allow you to observe that aspect of the medical student’s performance.

**1. Friendly communication**

( ) You did not greet me, or greeted me perfunctorily, or communicated with me rudely during the encounter.

( ) Your greeting and/or behavior during the encounter was generally polite but impersonal or distant.

( ) You greeted me warmly and communicated with me in a friendly, personal manner throughout the encounter.

( ) Your greeting and overall communication were friendly and compassionate. Overall, you created an exceptionally warm and friendly environment that made me feel comfortable to tell you all of my problems.

**2. Respectful treatment**

( ) You showed an obvious sign of disrespect during the encounter. E.g.: You treated me as an inferior.

( ) You did not show disrespect to me. However, I observed some signs of condescending behavior. Although I believe it was unintentional, it made me feel that I was not at the same level with you.

( ) You gave several indications of respecting me. If there was a physical exam, this includes draping me appropriately.

( ) You were exceptionally respectful throughout the encounter. Your verbal and nonverbal communication showed respect for my privacy, my opinions, my rights, and/or my socioeconomic status, etc..

**3. Listening to my story**

( ) You rarely gave me any opportunity to tell my story and/or frequently interrupted me while I was talking, not allowing me to finish what I said. Sometimes I felt you were not paying attention (for example, you asked for information that I already provided).

( ) You let me tell my story without interruption, or only interrupted appropriately and respectfully. You seemed to pay attention to my story and responded to what I said appropriately.

( ) You allowed me to tell my story without inappropriate interruption, responded appropriately to what I said, and asked thoughtful questions to encourage me to tell more of my story.

( ) You were an exceptional listener. You encouraged me to tell my story and checked your understanding by restating important points.

**4. Interest in me as a person.**

( ) You never showed interest in me as a person. You only focused on the disease or medical issue.

( ) In addition to talking about my medical issue, you spent some time getting to know me as a person.

( ) You spent some time exploring how my medical issue affects my personal or social life.

( ) You were exceptionally interested in me as a person. You not only explored how my medical problem affects my personal and social life, but also showed your willingness to help me address those challenges.

**5. Encouraging my questions**

( ) You did not solicit questions, or frequently avoided my questions, or did not provide helpful answers.

( ) You sometimes asked if I had questions, but seldom waited at least 5 seconds to allow me to formulate questions. You addressed my questions briefly without avoiding them.

( ) You actively encouraged me to ask questions, paused to allow me to formulate them, and provided clear and sufficient answers to all of my questions.

( ) You actively encouraged me to ask questions several times during the encounter, with sufficient wait time. You spent significant time and effort to answer my questions clearly and confirmed that I understood the answer and that my concerns were addressed.

**6. Physical examination**

( ) You never or rarely warned me about what you were going to do with my body. You also never or rarely explained what you found from the physical examination.

( ) You did not warn me about what you were going to do with my body, OR did not explain to me pertinent findings (both negative and positive) from your physical examination.

( ) You told me what you were going to do to my body AND described what you found.

( ) You helped me understand clearly what you were going to do to my body. You also provided clear explanation of what you found from the physical examination and the implications of your findings for my situation.

( ) **Not applicable**. There was no physical examination in this case.

**7. Appropriate vocabulary**

( ) You used vocabulary that was too simple or too complex for me, or frequently used medical terms without explaining them to me. Sometimes I could not understand what you said to me without asking for explanations of terms you used.

( ) Your vocabulary was generally appropriate but you sometimes inadvertently used medical terms without explaining them to me.

( ) Your vocabulary was appropriate and if needed you provided brief explanations of any medical terms you used without my prompting.

( ) Your vocabulary was appropriate and you always provided clear and full explanation of relevant medical terms you used. In addition, you helped me better my understanding of my condition with the medical terms you explained to me.

**8. Sensitive subject matters** (e.g., sexual history, tobacco/alcohol/drug use, religious/cultural issues, giving bad news, or difficult emotional states)

( ) You never warned me before approaching sensitive subject matters. You seemed judgmental and clearly expressed your disapproval of my positions or feelings, making me feel uncomfortable about discussing these subjects or feelings with you.

( ) You were careful and nonjudgmental in discussing sensitive subject matters. However, you did not express understanding of my feelings and did not provide much emotional support.

( ) You were sensitive about discussing difficult subjects and were respectful of my feelings. I never sensed that you were judgmental or disapproving of my positions or feelings on these subjects. You showed empathic understanding of my position or feelings and provided appropriate emotional support.

( ) You were unusually empathic, sensitive and respectful of me and of my feelings and provided exceptional emotional support. In addition, you verbally reflected these back to me (e.g., “You sound sad”) to show your understanding.

( ) **Not applicable**. There were no sensitive subject matters in this case.

**9. Closing the encounter**

( ) You ended the session abruptly without discussion of next steps or follow up.

( ) You briefly explained what to expect next, but left out essential elements such as a summary of the session and your assessment, the timeline for next steps, and/or asking if I had any questions.

( ) You summarized the session and your assessment and fully clarified next steps. You asked if I had any questions about the plan.

( ) In addition to summarizing the session and clarifying plans, you provided a safety net by explaining possible unexpected outcomes and when and how to seek help, and/or asked about any possible barriers to the plan, and/or affirmed my agreement and commitment to the plan.

**10. Do I want to see you again as a member of my healthcare team?**

( ) I did not feel comfortable in communicating with you at all. I would rather not have you on my team.

( ) I think you were okay in general and might let you see me again.

( ) I was impressed by the way you communicated with me. I would like to see you again.

( ) I was very impressed with you. I think you are one of the best medical students I have ever seen. I would feel very comfortable discussing any medical problems with you.

**Please add additional comments here:**

**EMR RELATED PRO/COM QUESTIONS**

1. When entering the room, how did the student start the visit in regard to the computer and the electronic health record (EMR)?
   1. The student immediately went to the computer and started opening the EMR before greeting me; I felt completely ignored.
   2. The student seemed distracted by his/her need to get to the computer; I felt like the computer took priority over me.
   3. The student briefly introduced him/herself first but then immediately went to the computer.
   4. The student was completely focused on me during the introduction and use of the computer came later in the visit.
   5. Did not integrate electronic health record during visit
2. How was the student in asking permission and/or explaining the purpose of using the EMR during the visit?
   1. The student did not ask my permission nor explain the purpose of using the EMR at all.
   2. The student either asked my permission to use the EMR or explained the purpose of using the EMR during our visit.
   3. The student asked my permission to use the EMR and explained the purpose of using the EMR during our visit, but did so only after I asked him/her what she was doing.
   4. The student asked my permission to use the EMR and explained the purpose of using the EMR during our visit before logging on.
   5. Did not integrate electronic health record during visit
3. How smoothly did the student use the EMR while in the room?
   1. The student spent most of his/her time on the computer and used that as a resource instead of me.
   2. The student spent a fair amount of time on the computer but still asked me for some information about my history.
   3. The student spent a little bit of time on the computer but did not seem to incorporate that information well when talking to me.
   4. The student spent a little bit of time on the computer and did so in a way that enhanced our communication and understanding my story.
   5. Did not integrate electronic health record during visit

1. How was the student at maintaining complete focus on you rather than the EMR during any emotionally sensitive moments in the interaction?
   1. The student seemed entirely focused on the computer and not me or my concerns/emotions.
   2. The student seemed distracted by the computer and missed some opportunities to address my concerns/emotions.
   3. The student primarily focused on my concerns and emotions but on occasion seemed more focused on the computer.
   4. The student was able to address my emotions and concerns without being distracted by the computer.
   5. No emotional/sensitive interaction opportunities arose during visit

1. How familiar did the student seem with regard to your health history to facilitate focused gathering of information during the visit?
   1. The student did not at all seem familiar with my history.
   2. The student seemed somewhat familiar with my history although I did have to correct or clarify key elements of my history.
   3. The student seemed mostly familiar with my history and required only a few corrections.
   4. The student seemed very familiar with my history.

**Student Instructions on using the EMR:**

The students are instructed to review your allergies and medications from information gleaned in his or her review of the Sally Martin file in the EMR.

They are also being instructed to review this information with you and then click “reviewed” on both items in the EMR – during the course of your interaction.
