# Supplementary material for: The Electronic Health Record Objective Structured Clinical Examination Station: Assessing Student Competency in Patient Notes and Patient Interaction
Source: MedEdPORTAL. 2020 Oct 28;16:10998. doi: 10.15766/mep_2374-8265.10998 (PMC7597945; doi:10.15766/mep_2374-8265.10998)
Supplement: Supplementary file 1 — EHR OSCE Introduction Video Script.docxOSCE SP Training Guide.docxOSCE Exam Case Summary Sheet.docxOSCE Patient Note Template.docxOSCE SP Postencounter Checklist.docxOSCE Patient Note Faculty Grading Rubric.docxEHR SP Case.docx [file mep_2374-8265.10998-s001.zip › C. OSCE Exam Case Summary Sheet.docx]

**Appendix C - OSCE exam case summary sheet**

Clinical Performance Examination

**A. Opening Scenario**

Mrs. Sally Martin is a 43-year-old woman who presents to the Family Medicine Clinic for a follow-up on her diabetes management.

**B. Vital Signs**

Blood Pressure 152/85

Temperature 98.7 °F

Pulse 60

Respiration Rate 16

POCT blood sugar 288

SpO2 98% RA

**C. Examinee Tasks**

1. Obtain a focused history. For the station, you are required to use the EHR to verity the patient’s allergies and medications.

2. Perform a relevant physical examination.

**(If a breast, pelvic/genital or rectal exam would be important to perform in this case, state so to the patient, but DO NOT perform the examination)**

3. Discuss your impressions and any recommendations

4. After leaving the room, complete your patient note on the computer
