# Supplementary material for: The Electronic Health Record Objective Structured Clinical Examination Station: Assessing Student Competency in Patient Notes and Patient Interaction
Source: MedEdPORTAL. 2020 Oct 28;16:10998. doi: 10.15766/mep_2374-8265.10998 (PMC7597945; doi:10.15766/mep_2374-8265.10998)
Supplement: Supplementary file 1 — EHR OSCE Introduction Video Script.docxOSCE SP Training Guide.docxOSCE Exam Case Summary Sheet.docxOSCE Patient Note Template.docxOSCE SP Postencounter Checklist.docxOSCE Patient Note Faculty Grading Rubric.docxEHR SP Case.docx [file mep_2374-8265.10998-s001.zip › D. OSCE Patient Note Template.docx]

**Appendix D: OSCE patient note template**

| HISTORY: Describe the history you just obtained from this patient. Include only information (pertinent positives and negatives) relevant to this patient's problem(s). |  |
| --- | --- |
|  |  |
| PHYSICAL EXAMINATION: Describe any positive and negative findings relevant to this patient's problem(s). Be careful to include only those parts of examination you performed in this encounter. |  |
| DATA INTERPRETATION: Based on what you have learned from the history and the physical examination, list up to 3 diagnoses that might explain this patient's complaint(s). List your diagnoses from most to least likely. For some cases, fewer than 3 diagnoses will be appropriate. Then, enter the positive or negative findings from the history and the physical examination (if present) that support each diagnosis. Lastly, list initial diagnostic studies (if any) you would order for each listed diagnosis (e.g. restricted physical exam maneuvers, laboratory tests, imaging, ECG, etc.) |  |
| Diagnosis #1   \| History Finding(s) \| Physical Exam Finding(s) \| \| --- \| --- \| \|  \|  \| \|  \|  \| \|  \|  \| |  |
| Diagnosis #2   \| History Finding(s) \| Physical Exam Finding(s) \| \| --- \| --- \| \|  \|  \| \|  \|  \| \|  \|  \| |  |
| Diagnosis #3   \| History Finding(s) \| Physical Exam Finding(s) \| \| --- \| --- \| \|  \|  \| \|  \|  \| \|  \|  \| |  |
| \| Diagnostic Study/Studies \| \| --- \| |  |
