# Supplementary material for: The Electronic Health Record Objective Structured Clinical Examination Station: Assessing Student Competency in Patient Notes and Patient Interaction
Source: MedEdPORTAL. 2020 Oct 28;16:10998. doi: 10.15766/mep_2374-8265.10998 (PMC7597945; doi:10.15766/mep_2374-8265.10998)
Supplement: Supplementary file 1 — EHR OSCE Introduction Video Script.docxOSCE SP Training Guide.docxOSCE Exam Case Summary Sheet.docxOSCE Patient Note Template.docxOSCE SP Postencounter Checklist.docxOSCE Patient Note Faculty Grading Rubric.docxEHR SP Case.docx [file mep_2374-8265.10998-s001.zip › E. OSCE SP Postencounter Checklist.docx]

**Appendix E - OSCE standardized patient post encounter checklist**

History Checklist

| Did the student elicit subjective information and/or confirm Electronic Health Record Data regarding: | |
| --- | --- |
| Recent blood sugar readings | ( ) Yes |
|  | ( ) No |
| Polyuria, polydipsia, or increased thirst (any one of the three) | ( ) Yes |
|  | ( ) No |
| Change in appetite or diet (either is acceptable) | ( ) Yes |
|  | ( ) No |
| Hypoglycemic Symptoms | ( ) Yes |
|  | ( ) No |
| Confirm insulin dose and timing | ( ) Yes |
|  | ( ) No |
| Confirm oral steroid (prednisone) date of end of prescription | ( ) Yes |
|  | ( ) No |
| Upper respiratory symptoms associated with recent Urgent Care Visit | ( ) Yes |
|  | ( ) No |
| Past Medical History/Health | ( ) Yes |
|  | ( ) No |
| Confirm patient's other medications against Electronic Health Record | ( ) Yes |
|  | ( ) No |

Physical Exam Checklist

| Did the student perform the following maneuvers: | |
| --- | --- |
| Washed hands before touching the patient | ( ) Yes |
|  | ( ) No |
| Auscultation of heart sounds | ( ) Yes |
|  | ( ) No |
| Peripheral sensory neurologic exam | ( ) Yes |
|  | ( ) No |
| Student used stethoscope directly on the skin for both all auscultations NOT through gown | ( ) Yes |
|  | ( ) No |

Communication and Professionalism

| Please choose the option that best describes how you feel toward the medical student’s communication skills. Some items also have a ‘not applicable’ option. Select this option when the context of the case does not allow you to observe that aspect of the medical student’s performance. | | | |
| --- | --- | --- | --- |
| Friendly Communication | ( ) You did not greet me, or greeted me perfunctorily, or communicated with me rudely during the encounter | | |
|  | ( ) Your greeting and/or behavior during the encounter was generally polite but impersonal or distant | | |
|  | ( ) You greeted me warmly and communicated with me in a friendly, personal manner throughout the encounter | | |
|  | ( ) Your greeting and overall communication were friendly and compassionate. Overall, you created an exceptionally warm and friendly environment that made me feel comfortable to tell you all of my problems | | |
| Comments: | | | |
| Respectful treatment | ( ) You showed an obvious sign of disrespect during the encounter, for example, you treated me as an inferior | | |
|  | ( ) You did not show disrespect to me. However, I observed some signs of condescending behavior. Although I believe it was unintentional, it made me feel that I was not at the same level with you | | |
|  | ( ) You gave several indications of respecting me. If there was a physical exam, this includes draping me appropriately | | |
|  | ( ) You were exceptionally respectful throughout the encounter. Your verbal and nonverbal communication showed respect for my privacy, my opinions, my rights, and/or my socioeconomic status | | |
| Comments: | | | |
| Listening to my story | ( ) You rarely gave me any opportunity to tell my story and/or frequently interrupted me while I was talking, not allowing me to finish what I said. Sometimes I felt you were not paying attention (for example, you asked for information that I already provided). | | |
|  | ( ) You let me tell my story without interruption, or only interrupted appropriately and respectfully. You seemed to pay attention to my story and responded to what I said appropriately | | |
|  | ( )  You allowed me to tell my story without inappropriate interruption, responded appropriately to what I said, and asked thoughtful questions to encourage me to tell more of my story | | |
|  | ( ) You were an exceptional listener. You encouraged me to tell my story and checked your understanding by restating important points | | |
| Comments: | | | |
| Interest in me as a person | ( ) You never showed interest in me as a person. You only focused on the disease or medical issue | | |
|  | ( )  In addition to talking about my medical issue, you spent some time getting to know me as a person | | |
|  | ( ) You spent some time exploring how my medical issue affects my personal or social life | | |
|  | ( ) You were exceptionally interested in me as a person. You not only explored how my medical problem affects my personal and social life, but also showed your willingness to help me address those challenges | | |
| Comments: | | | |
| Encouraging my questions | ( ) You did not solicit questions, or frequently avoided my questions, or did not provide helpful answers | | |
|  | ( ) You sometimes asked if I had questions, but seldom waited at least 5 seconds to allow me to formulate questions. You addressed my questions briefly without avoiding them | | |
|  | ( ) You actively encouraged me to ask questions, paused to allow me to formulate them, and provided clear and sufficient answers to all of my questions. | | |
|  | ( ) You actively encouraged me to ask questions several times during the encounter, with sufficient wait time. You spent significant time and effort to answer my questions clearly and confirmed that I understood the answer and that my concerns were addressed | | |
| Comments: | | | |
| Physical examination | ( )  You never or rarely warned me about what you were going to do with my body. You also never or rarely explained what you found from the physical examination. | | |
|  | ( )  You did not warn me about what you were going to do with my body, OR did not explain to me pertinent findings (both negative and positive) from your physical examination | | |
|  | ( ) You told me what you were going to do to my body AND described what you found | | |
|  | ( ) You helped me understand clearly what you were going to do to my body. You also provided clear explanation of what you found from the physical examination and the implications of your findings for my situation | | |
|  | ( ) Not applicable. There was no physical examination in this case | | |
| Comments: | | | |
| Appropriate vocabulary | ( ) You used vocabulary that was too simple or too complex for me, or frequently used medical terms without explaining them to me. Sometimes I could not understand what you said to me without asking for explanations of terms you used | | |
|  | ( ) Your vocabulary was generally appropriate but you sometimes inadvertently used medical terms without explaining them to me | | |
|  | ( ) Your vocabulary was appropriate and if needed you provided brief explanations of any medical terms you used without my prompting | | |
|  | ( ) Your vocabulary was appropriate and you always provided clear and full explanation of relevant medical terms you used. In addition, you helped me better my understanding of my condition with the medical terms you explained to me | | |
| Comments: | | | |
| Sensitive subject matters (e.g., sexual history, tobacco/alcohol/drug use, religious/cultural issues, giving bad news, or difficult emotional states) | ( ) You never warned me before approaching sensitive subject matters. You seemed judgmental and clearly expressed your disapproval of my positions or feelings, making me feel uncomfortable about discussing these subjects or feelings with you | | |
|  | ( ) You were careful and nonjudgmental in discussing sensitive subject matters. However, you did not express understanding of my feelings and did not provide much emotional support | | |
|  | ( ) You were sensitive about discussing difficult subjects and were respectful of my feelings. I never sensed that you were judgmental or disapproving of my positions or feelings on these subjects. You showed empathic understanding of my position or feelings and provided appropriate emotional support | | |
|  | ( ) You were unusually empathic, sensitive and respectful of me and of my feelings and provided exceptional emotional support. In addition, you verbally reflected these back to me (e.g., “You sound sad”) to show your understanding | | |
|  | ( ) Not applicable. There were no sensitive subject matters in this case   (NS) | | |
| Comments: | | | |
| Closing the encounter | ( ) You ended the session abruptly without discussion of next steps or follow up. | | |
|  | ( ) You briefly explained what to expect next, but left out essential elements such as a summary of the session and your assessment, the timeline for next steps, and/or asking if I had any questions | | |
|  | ( ) You summarized the session and your assessment and fully clarified next steps. You asked if I had any questions about the plan | | |
|  | ( ) In addition to summarizing the session and clarifying plans, you provided a safety net by explaining possible unexpected outcomes and when and how to seek help, and/or asked about any possible barriers to the plan, and/or affirmed my agreement and commitment to the plan | | |
| Comments: | | | |
| Do I want to see you again as a member of my healthcare team | ( ) I did not feel comfortable in communicating with you at all. I would rather not have you on my healthcare team | | |
|  | ( ) I think you were okay in general and might let you see me again | | |
|  | ( ) I was impressed by the way you communicated with me. I would like to see you again | | |
|  | ( ) I was very impressed with you. I think you are one of the best medical students I have ever seen. I would feel very comfortable discussing any medical problems with you | | |
| Comments: | | | |
| When entering the room, how did the student start the visit in regard to the computer and the electronic health record (EMR)? | ( ) The student immediately went to the computer and started opening the EMR before greeting me; I felt completely ignored. | | |
|  | ( ) The student seemed distracted by his/her need to get to the computer; I felt like the computer took priority over me. | | |
|  | ( ) The student briefly introduced him/herself first but then immediately went to the computer | | |
|  | ( ) The student was completely focused on me during the introduction and use of the computer came later in the visit | | |
|  | ( )  Did not integrate electronic health record during visit | | |
| How was the student in asking permission and/or explaining the purpose of using the EMR during the visit? | ( ) The student did not ask my permission nor explain the purpose of using the EMR at all. | | |
|  | ( ) The student either asked my permission to use the EMR or explained the purpose of using the EMR during our visit. | | |
|  | ( ) The student asked my permission to use the EMR and explained the purpose of using the EMR during our visit, but did so only after I asked him/her what she was doing. | | |
|  | ( ) The student asked my permission to use the EMR and explained the purpose of using the EMR during our visit before logging on. | | |
|  | ( ) Did not integrate electronic health record during visit | | |
| How smoothly did the student use the EMR while in the room? | ( ) The student spent most of his/her time on the computer and used that as a resource instead of me | | |
|  | ( ) The student spent a fair amount of time on the computer but still asked me for some information about my history | | |
|  | ( ) The student spent a little bit of time on the computer but did not seem to incorporate that information well when talking to me | | |
|  | ( ) The student spent a little bit of time on the computer and did so in a way that enhanced our communication and understanding my story | | |
|  | ( ) Did not integrate electronic health record during visit | | |
| How was the student at maintaining **complete focus on you** rather than the EMR during any emotionally sensitive moments in the interaction? | ( ) The student seemed entirely focused on the computer and not me or my concerns/emotions | | |
|  | ( ) The student seemed distracted by the computer and missed some opportunities to address my concerns/emotions | | |
|  | ( ) The student primarily focused on my concerns and emotions but on occasion seemed more focused on the computer | | |
|  | ( ) The student was able to address my emotions and concerns without being distracted by the computer | | |
|  | ( ) No emotional/sensitive interaction opportunities arose during visit | | |
| How familiar did the student seem with regard to your health history to facilitate focused gathering of information during the visit? | ( )  The student did not at all seem familiar with my history. | | |
|  | ( ) The student seemed somewhat familiar with my history although I did have to correct or clarify key elements of my history | | |
|  | ( ) The student seemed mostly familiar with my history and required only a few corrections | | |
|  | ( ) The student seemed very familiar with my history | | |
| Additional Comments (Please include any additional comments. Please be as specific as possible without giving away case details). | | | |
|  | |  |  |

Bottom of Form
