# Supplementary material for: The Electronic Health Record Objective Structured Clinical Examination Station: Assessing Student Competency in Patient Notes and Patient Interaction
Source: MedEdPORTAL. 2020 Oct 28;16:10998. doi: 10.15766/mep_2374-8265.10998 (PMC7597945; doi:10.15766/mep_2374-8265.10998)
Supplement: Supplementary file 1 — EHR OSCE Introduction Video Script.docxOSCE SP Training Guide.docxOSCE Exam Case Summary Sheet.docxOSCE Patient Note Template.docxOSCE SP Postencounter Checklist.docxOSCE Patient Note Faculty Grading Rubric.docxEHR SP Case.docx [file mep_2374-8265.10998-s001.zip › F. OSCE Patient Note Faculty Grading Rubric.docx]

**Appendix F - OSCE patient note faculty grading rubric**

**History**

Did student note document the following (yes or no):

| 1.Recent blood sugar readings  (1 point) | ( ) Yes |
| --- | --- |
|  | () No |
| 2.Symptoms of hyperglycemia including polyuria and/or polydipsia and/or increased thirst  (1 point) | ( ) Yes |
|  | () No |
| 3.Change in appetite or recent dietary habits  (1 point) | ( ) Yes |
|  | ( ) No |
| 4.Hypoglycemic symptoms  (1 point) | ( ) Yes |
|  | (x) No |
| 5.Record patient’s insulin dose and timing of administration  (1 point) | ( ) Yes |
|  | () No |
| 6.Record patient taking prednisone  (1 point) | ( ) Yes |
|  | ( ) No |
| 7.Brief description of recent upper respiratory symptoms  (1 point) | ( ) Yes |
|  | ( ) No |
| 8. Past Medical History  (1 point) | ( ) Yes |
|  | ( ) No |
| 9. Medications (1 point) | ( ) Yes |
|  | ( ) No |

**Physical Exam**

| Did did student note document the following (yes or no): | |
| --- | --- |
| 1.Vital Signs (1 point) | ( ) Yes |
|  | ( ) No |
| 2.Body habitus and/or other general observations (1 point) | ( ) Yes |
|  | ( ) No |
| 3. Auscultatory Lung Exam Findings (1 point) | ( ) Yes |
|  | ( ) No |
| 4. Peripheral lower extremity neurologic exam findings (1 point) | ( ) Yes |
|  | ( ) No |

**Data Integration**

| Assessment/Differential Diagnosis for DM | |
| --- | --- |
| Did the student include any of the following diagnoses  (1 point) | ( ) Diabetes with semantic qualifier: described as worsening, poorly controlled or uncontrolled (the student's impression is not the key element here but rather that they describe the current state of the patient's diabetes: could be say "stable" or more likely "worsening," etc) |
|  | ( ) Inclusion of a suspected etiology or trigger of worsening diabetes: steroid induced hyperglycemia, dietary fluctuation/non-adherence, acute illness associated hyperglycemia, and/or chronic disease progression |
|  | ( ) Inclusion of subjective and/or objective PE/lab data to support clinical reasoning (for example, student may cite patient's reported recent blood sugars; student may also acknowledge symptoms of nocturia/polyuria as sugestive of poor blood sugar control) |
|  | ( ) Recognition of impact of steroids and acute illness on blood glucose/DM control (student acknowledges that the patient is on steroids and this is contibutory to the patient's hyperglycemia) |
|  | ( ) Recognition of end organ impact of patient’s diabetes: neuropathy and/or CKD |
|  | ( )  None of the above |

**Diagnostic Study/Studies**

| Plan and Management for DM | |
| --- | --- |
| 1. Includes specifically addressing insulin management (1 point) | ( ) Yes |
|  | () No |
| 1. Includes justification for insulin management strategy (1 point) | ( ) Yes |
|  | () No |
| 1. Includes obtaining HbA!C to assess (1 point) | () Yes |
|  | ( ) No |
| 1. Plan acknowledges dietary considerations in diabetes management (1 point) | () Yes |
|  | ( ) No |
| 1. Disposition and timing for return to clinic included (1 point) | ( ) Yes |
|  | () No |

Please provide overall comments for the student regarding the case
