# Supplementary material for: The Electronic Health Record Objective Structured Clinical Examination Station: Assessing Student Competency in Patient Notes and Patient Interaction
Source: MedEdPORTAL. 2020 Oct 28;16:10998. doi: 10.15766/mep_2374-8265.10998 (PMC7597945; doi:10.15766/mep_2374-8265.10998)
Supplement: Supplementary file 1 — EHR OSCE Introduction Video Script.docxOSCE SP Training Guide.docxOSCE Exam Case Summary Sheet.docxOSCE Patient Note Template.docxOSCE SP Postencounter Checklist.docxOSCE Patient Note Faculty Grading Rubric.docxEHR SP Case.docx [file mep_2374-8265.10998-s001.zip › G. EHR SP Case.docx]

Appendix G: Standardized Patient Case

Date: 9/24/2019

Primary Case Author: Joseph A. Cristiano M.D.

Secondary Case Author: N/A

Standardized Patient Educator: Leslie R. Ellis M.D.; Andrea Vallevand Ph.D.

Name of Case: Sally Martin

Name of educational and or assessment activity: OSCE EMR Case

Patient Name: Sally Martin

Chief Complaint: This is a follow up appointment on diabetes management

Most likely Diagnosis and Differential with rationale from history and/or physical exam:

Diabetes

Challenge question: N/A

Domains: Check all that apply

- Professionalism

× Communication and Interpersonal skills

× Medical History

× Physical exam

- Shared Decision Making
- Patient Education

× Clinical Reasoning

× Documentation

- Handoff
- Presentation
- Other:

Type and level of learner: Undergraduate Medical Student; resident

Case Objectives: please list specific objectives for each of the domains you have checked above:

1. Demonstrate empathic and patient centered communication skills with use EHR during a patient encounter.

2. Perform a focused history and physical exam for the evaluation of a patient presenting for follow up related to diabetes.

3. Write an electronic patient care note in SOAP format that is clinically reasoned and effectively communicates key elements of history, physical exam and the assessment and plan.

| SETTING: outpatient, in patient, ED, home, nursing home, rehab, group etc. | Outpatient |
| --- | --- |
| PATIENT PROFILE: Information about the “patient” that helps select an SP and helps the learner get an understanding of them as a person. SP will know more information about the patient than learner will ever ask but allows SP to portray a fully developed patient personality. If none of the items below are particulars for the case please write “all may be used.” | |
| Age range | 43 year old |
| Religious/spiritual background | n/a |
| Sex (e.g., male, female, intersex, transwoman, transman) | female |
| Sexual Orientation (e.g., heterosexual, lesbian, gay, bisexual, pansexual, queer, asexual) | heterosexual |
| Gender expression (e.g., man, woman, gender queer) | woman |
| Race/ethnicity: | Can be any ethnicity |
| Physical description (e.g., BMI, height range) | middle age slightly overweight |
| Physical limitations | None |
| Patient appearance (e.g., disheveled, hospital gown, business casual, casual) | well appearing |
| Moulage + location (e.g., none, bruises, scars, body piercing, tattoos) | None |
| Affect (e.g., pleasant, cooperative) | Friendly, calm but concerned about her diabetes management and increase in blood sugars. Is somewhat overwhelmed about recent illness and increase in number of medications recently prescribed |
| Family group (e.g., who is family, who they live with) | Married and lives with husband and daughter. |
| Education | High school |
| Level of health literacy | High |
| Employment, if any - present and past, noting any current stresses | Office clerk |
| Home/homeless - type of dwelling, number of stories, owned or rented | Married and lives with husband and daughter. Own family |
| Financial situation- any current stresses | Stable |
| Insurance Status (e.g., un/under/insured, public/private, HMO/PPO) | Insured |
| Habits (i.e., diet, exercise, caffeine, smoking, alcohol, drugs) | Smoker 1 pack per day for 27 years |
| Activities (i.e., hobbies, sports, clubs, friends) | does not exercise – but has signed up for a hospital-sponsored “Couch to 5K walk” program |
| Typical day - what is the usual daily routine | The patient is sedentary and recognizes that there is area to improve her health. She sits most of day while working. She does not exercise. |

| CASE INFORMATION | |
| --- | --- |
| Chief Concern: What the patient will say when greeted by the student. The patient’s primary reason for seeking medical care often stated in his/own words. | She is concerned about her diabetes and she suspects it is not well controlled. |
| Additional Concerns: Other, if any, concerns the patient has today (i.e., symptoms, requests, expectations, etc.) that will become part of set agenda. | She is concerned about her recent respiratory infection, especially related to being treated with steroids and the effect on her diabetes |
|  | |
| THE PATIENT STORY: The SP will be asked to tell their symptom story and the personal and emotion impact for each of their concerns. You will want to write this is the patient voice. The symptom story should be able to answer this question: “Tell me more about [chief concern/additional concern], starting at the beginning and bringing me up to now.”  The personal context should be able to answer questions concerning the broader personal/psychosocial context of symptoms, especially the patient beliefs/attributions.  The emotional context should be able to ask how are you doing with this, how does this make you feel, how has this affected you emotionally? IMPACT: How has this affected your life? How has this been for your family? | Your opening line:  “I’m here for a follow-up on my diabetes management”  How you will present yourself to the student:   - You are friendly, however you will express concern about your health - You are feeling overwhelmed about your recent health set-back and with all the additional medications that were prescribed (for your “cold” – information below) - You are also overwhelmed about the increase in blood sugar readings, recently (information below) - You are compliant with all your medications, but you really do not have a strong understanding of your diabetes management   Diabetes history:   - Your PCP diagnosed you with Type II diabetes approximately 3 years ago. Took an oral medication for two years, but it wasn’t working. If asked, you don’t remember the name, but it started with an “M”. If the student says “Metformin” – you say “yes”. - Your PCP started you on insulin 9 months ago at 30 units twice daily. - You attended a follow-up appointment 6 months ago when your PCP increased your insulin 40 units twice daily. - You missed your follow up appointment 3 months ago due to a “fender-bender” on your way to the clinic (nobody was hurt, but the front wheel was too damaged to drive on). - You are currently taking 40 units of insulin twice a day.   Your blood sugar readings have normally been 160 to 200, but recently they have been in the 240-260s. You will wonder if being sick and being on the medications you were prescribed for your “cold” are the reason. |
| HISTORY OF PRESENT ILLNESS: Although some of the HPI will be given in the patient’s symptom story, the learners will expand the story during the direct question section. Below describe the detailed history, usually about the chief concern, which the student must develop in order to make a useful assessment of the problem: | |
|  | |
| Onset (when; gradual or sudden) | In the past several days blood sugar is 240 to 260, since onset of your respiratory illness and steroid administration |
| Setting (what was going on or where was patient when symptoms first noticed?) | Since the onset of a recent respiratory illness about a week ago, you have noticed the increase in blood sugar |
| Duration (how long) | Blood sugars have still been elevated since her recent office visit 6 months ago, but even prior to the illness they were not at her blood sugar goal (160-180 in the mornings and 200 mid-day). |
| Time relationships (frequency, constant or intermittent) | You take your blood sugar typically in the early morning before breakfast, but periodically you’ll take it mid-day (this is lunch time). Blood sugars in the mornings are usually around 240 and the mid-day readings are typically slightly higher around 260. |
| Location | n/a |
| Radiation | n/a |
| Quality | n/a |
| Amount | n/a |
| Aggravated by what | Aggravating/Alleviating Factors: You have been taking prednisone (a steroid) since an office visit 4 days ago for a respiratory infection and you’ve noticed your blood sugars increase since then. Prior to that time, your blood sugars in the morning were typically 160-180 and around 200 mid-day. |
| Relieved by what |  |
| Associated with what | Waking up in the middle of the night less so due to coughing now, but rather to urinate. You have been thirsty over the past few days as well and have not felt “yourself” in weeks. |
| Attitude (what does the patient think is the problem, and how does he/she feel about it) | Having to take steroids has been discouraging by doctors because you know it is worsening your diabetes and your blood sugars are higher. You also are concerned partly since your new PCP who you established care with 9 months ago emphasize the need to better control your diabetes. You also recognize that you need to quit smoking but there has been so many other things going on between your health specific to diabetes, your recent illness and home/work that you have not been able to address this. |
| Overall course |  |
| REVIEW OF SYSTEMS: Significant positives and negatives | |
|  | a. HEENT: Several months ago, you noticed some numbness in the bottom of your feet and burning. It seems to be improving since you started insulin.  b. Respiratory: you have a chronic morning cough that usually clears after a few minutes in the morning. You have been coughing more frequently with a recent respiratory infection but as characterized above it is improving and you are nearly back to your “baseline”  c. Cardiovascular (including peripheral): You deny any orthopnea (shortness of breath lying down). You do not notice leg swelling or weight gain recently.  d. GI: You sometimes get a bit nauseous after eating but no vomiting. Seems to help to eat smaller portions more frequently. Your appetite has been diminished for several months but had been improving in the past several weeks since starting insulin. More recently, your appetite has declined again with the respiratory illness. You deny any abdominal pain, diarrhea, blood in stool or tarry/dark stools.  e. GU: You have experienced increasing urination since you started steroids. You wake up at night 3-4 times to urinate and you urinate every 2-3 hours during the day. You deny any symptoms of retention of urine after voiding. You did notice some urinary incontinence with forceful coughing while you were sick but that has resolved.  f. Musculoskeletal: You deny any joint pains, but you do feel like you are just “not as strong” as you once were when you were younger (20’s and 30’s). No specific area and you feel weak overall since getting sick. Does not impact your ability to walk or do routine household/work duties.  g. Psychiatric: You are nervous about your health, but your mood is positive. You deny any depression, SI, HI, etc  Hematologic: You deny any easy bruising, bleeding or any other symptoms. |
|  | |
| Past medical history |  |
| Medication allergies (Name and reaction) | sulfa medications cause rash with hives |
| Environmental allergies (Name and reaction) | N/A |
| Illnesses | She also has been told she has “kidney problems” from her diabetes and high blood pressure as well as “COPD” from smoking. |
| Vaccinations | N/A |
| Surgeries | Laparoscopic cholecystectomy in 2012 |
| Accidents/ injuries/ trauma | No accident |
| Hospitalization | Only hospitalizations for for birth of son and daughter. No medical hospitalizations. |
|  | |
| Inclusive sexual and reproductive history | |
| Sexual practices  Sexual partners  Protection: Use of safer sex practices  Use of birth control if appropriate  Risk of intimate partner violence | Monogamous, Married and lives with husband and daughter |
| Ob/GYN HISTORY | Not included |
| Medications | Albuterol as required (prescribed as a “rescue inhaler” for COPD and also used for the cold)  Advair inhaled twice day 1 puff (for the COPD)  Spiriva inhaled once a day one puff (pill crushed in inhaler) (COPD)  Novolin N 40 units twice a day with breakfast and dinner (Diabetes)  Losartan (kidney problems and high blood pressure)  Simvastatin (cholesterol)  Azithromycin antibiotic added at urgent care visit for your “cold” (finishing tomorrow or day after)  Prednisone (oral steroid) added at urgent care visit for your “cold” (finishing tomorrow or day after) |
| Immunizations | × Tetanus  × Flu  × Hepatitis   - Pneumovax - HPV - Other |
| Tobacco products:  × Cigarettes   - Cigar - Pipe - Chew - E-cigarettes | - Never - Past- year started/year quit - Current   - 1 pack per day   - 27 of years |
| Alcohol   - Beer - Wine - Liquor - Other | × Never   - Past- year started/year quit - Current   - Quantity   - # of years |
| Drugs   - Weed - Cocaine - Heroin - Meth - Other - IV - Inhalants - Other | × Never   - Past- year started/year quit - Current   - Quantity - # of years |
| Diet (describe) | you will be meeting a diabetes educator, later today, to work on diet changes – you want to better manage your diet and diabetes – you are excited about this. you have your plans – again, politely re-direct a student who persists in talking about this |
| Exercise (describe) | Do not exercise. but has signed up for a hospital-sponsored “Couch to 5K walk” program |
| List any other important social history or information important to this case | n/a |
| Family history |  |
| Mother, Father, Siblings, Grandparents, and other significant findings. | None. |
|  |  |
| Physical Exam- List exam maneuvers expected for this case and any abnormal findings that SP will simulate. (tenderness, hyper-hypo reflex, rebound, weakness etc. )   - Washed Hands before touching patient - Auscultation of heart sounds - Funduscopic Exam (student provides retinal image card) - Auscultation of breath sounds (student receives respiratory card) - Inspection of abdomen or general exam/body habitus (student will recieve card for truncal obesity based on performance of abdominal exam and/or general inspection of body habitus) - Peripheral sensory neurologic exam - Student used stethoscope directly on the skin for both respiratory system and heart sound auscultations | |
| PHYSICAL EXAM FINDINGS |  |
| 1. Written in layman’s terms | - 1. Gastrointestinal: normal   2. Skin: No skin problems.   3. Genitourinary: No exam required.   4. Eyes: No exam required   5. Hematologic: normal   6. Breasts: No exam is required as part of this visit   7. Musculoskeletal: normal muscle tone and bulk   8. Respiratory: clear to auscultation, end expiratory wheezes at the apices bilaterally (card to be presented to student if he or she listens to the lungs above the shoulder blades)   9. Peripheral Vascular: normal   10. Cardiovascular: normal   11. Neurologic: stocking paresthesia in lower extremities bilaterally in the feet (SP will portray)   12. Mental Status Exam: alert, oriented, conversant, no deficits |
| 1. General appearance- affect, appearance, position of patient at opening (i.e. sitting, laying down, holding abdomen etc.) | - Sitting - You are friendly, however you will express concern about your health |
| 1. Vital signs | - 1. BP: 152/85 mmHg   2. P: 60 BPM   3. Temp: 98.7F   4. Resp: 16   5. POCT blood sugar: 288   6. SpO2: 98% RA |
| 1. Specific findings and affect | N/A |
| 1. Response to certain physical movements | N/A |
|  |  |
| DIAGNOSIS AND DIFFERENTIAL |  |
| Diagnosis with support from positive and negative history and PE findings | Diagnosis is not part of this OSCE case. This is a diabetes management follow up visit. |
| Differential with support from positive and negative history and PE findings | Diagnosis is not part of this OSCE case. This is a diabetes management follow up visit. |
|  |  |
| MANAGEMENT OR DIAGNOSITIC PLAN | - Refer to dietician  - Set concrete goals  - Increase exercise, provide informational sheet that may help get the patient started  - Gain social support to work out with the patient  - Create log of blood glucose so we can monitor sugars while she is on the steroids  - Schedule follow up appointment |
|  |  |
| PROFESSIONALISM ISSUES OR CHALLENGES: | n/a |
